# Which procedures are performed by general internists practicing primary care in Germany? - a cross-sectional study

**DOI:** 10.1186/s12875-020-01136-7

**Published:** 2020-04-29

**Authors:** Kristina Flaegel, Bettina Brandt, Katja Goetz, Jost Steinhaeuser

**Affiliations:** 1 Institute of Family Medicine, University Hospital Schleswig-Holstein, Campus Lübeck, Ratzeburger Allee 160, 23538 Lübeck, Germany; 2 Department of General Practice/Primary Care, Hamburg University Medical School, Martinistraße 52, 20246 Hamburg, Germany

**Keywords:** Procedural skills, Residency training programs, Primary care, General internal medicine

## Abstract

**Background:**

Due to differences of residency training programs’ emphases – inpatient vs office-based – internal medicine and family medicine residents consistently reported differences in preparedness to care for common adult conditions. Study’s aim was to add knowledge about procedures that a) are performed by general internists working in primary care and b) should be learned during residency in general internists’ appraisal.

**Methods:**

A cross-sectional postal survey was carried out by using a questionnaire that comprised 90 procedures relevant in primary care. Each procedure implied the two questions “Do you perform this procedure in your own practice?” and “How important do you think it is to learn this procedure during residency?” The final questionnaire was sent to 1002 general internists working in primary care in Germany in May 2015. Data analysis was performed using SPSS Version 24.0 (SPSS inc., IBM). Next to descriptive statistics subgroup analyses were performed using cross tabulation and Chi-square tests for evaluation of differences in the performance of most frequently performed procedures in urban or rural areas as well as by male or female physicians.

**Results:**

Twenty-eight percent of sent questionnaires (276/1002) could be included in analysis. Mean age of participants was 52 years with 13 years of practice experience; 40% were female.

Twenty-nine (32%) of 90 given procedures were performed by at least half of the participants, foremost technical diagnostics, punctures, procedures of the integument and resuscitation. After Bonferroni correction, five of those procedures were performed by more male than female physicians and two procedures by more physicians working in a rural practice than physicians practicing in an urban location. Moreover, 46 (51%) procedures were assessed as important to learn during residency by at least 50% of participants.

**Conclusions:**

General internists working in German primary care perform a narrow scope of procedures offered by primary care physicians. In order to provide best ambulatory care for patients, residency training programs must ensure training in procedures that are necessary for providing high quality care. Therefore, a consensus aligned with patients’ and health-systems’ needs on procedures required for working as a general internist in primary care is necessary.

## Background

The World Health Organization (WHO) described distinctive features of primary care as person-centeredness, continuity, comprehensiveness and integration. All of these aspects contribute to quality of care and better outcomes [1–3].

Primary care is provided by specialists in family medicine, internal medicine or pediatrics “to the undifferentiated patient at the point of first contact” [4]. These physicians must be trained specifically in order to provide comprehensive primary care to all ages of patients, including acute, chronic and preventive care [4].

In Germany, the Association of Statutory Health Insurance Physicians (ASHIP) is in charge of need related planning. It regulates the regional primary care physician accreditation process comprising the restriction of physician accreditation due to oversupply and the maintenance of a sufficient supply of physicians otherwise [5]. By law, the accreditation process is in favor of specialists in family medicine rather than general internists [6]. Since there is a growing family physician shortage in Germany as in other countries worldwide [7–9], the number of general internists practicing primary care is steadily increasing [10, 11].

As of December 31, 2016, the number of primary care physicians (exclusive of pediatricians) in Germany added up to 54,604 [11] with 82.5 million inhabitants in need of primary care [12]. In total, 14,853 general internists practiced primary care implying that 27% of primary care were delivered by specialists in internal medicine [11].

Due to differences of residency training programs’ emphases – inpatient vs office-based – internal medicine and family medicine residents consistently reported differences in preparedness to care for common adult conditions [13]. From a health services research perspective, it is crucial to know by whom patients and their distinctive needs are cared for. This influences directly the emphases a residency training has to fulfill.

Primary care’s aim of comprehensiveness includes both the depth and breadth of conditions managed and the scope of services offered [14]. These services comprise evaluation and management services, test services as well as procedural services [2].

The aim of this study was to investigate, which procedures were performed by general internists working in primary care, and were important to learn during residency training in the appraisal of general internists.

## Methods

We carried out a cross-sectional study using a postal questionnaire sent to general internists practicing in primary care in Germany.

### Questionnaire

The 89-item-questionnaire’s development was based on international publications on core procedural skills [15–17] culturally adapted by German family physicians in a consensus process. The questionnaire was already sent to 1576 specialists in family medicine in Germany in 2012 [18]. Free text analysis of this study led to the inclusion of one more procedure (“audiometry”). Thus, the final questionnaire comprised 90 procedures relevant in the primary care setting. According to the definition developed by an Australian research team in consensus process procedures were defined as discrete, diagnostic or therapeutic activities performed on patients, requiring knowledge and manual skills [17]. The questionnaire was not validated for inter- and intra-rater reliability.

Each procedure implied the two questions: “Do you perform this procedure in your own practice?” (yes or no) and “How important do you think it is to learn this procedure during residency?” on a scale from 1 = very important to 4 = not important.

The questionnaire was structured for clear arrangement and analysis reasons in areas related to anatomical aspects (integument, eyes, ears, nose, chest, gastrointestinal tract, urogenital system, obstetrics and pediatrics, musculoskeletal system) as well as resuscitation, punctures and technical diagnostics. Nine items on sociodemographic data and a free text question on important procedures that had not been listed complemented the final questionnaire.

The final questionnaire is available as translated English version from Additional file 1.

### Participants

The addresses of general internists practicing in primary care were searched in the online registries of the federal states’ ASHIPs. These online registries display mainly practices’ contact details; some of them add further information on the physicians’ additional qualifications and practices’ services. Randomly, the federal states Lower Saxony, Rhineland Palatinate, Brandenburg and Saarland were chosen yielding in 1002 addresses of general internists practicing in primary care in these federal states. No further in- or exclusion criteria were formulated. We sent the questionnaire including a personalized study invitation and information sheet to all identified physicians with the initial deadline June 5, 2015 via regular mail. For respond enhancement one reminder with personalized study information and questionnaire was sent 6 weeks after first contact with the deadline July 31, 2015, again via regular mail. Pre-printed business reply envelopes were provided both times.

### Statistical analysis

Data management and analysis was performed using SPSS Version 24.0 (SPSS inc., IBM). Frequencies and percentages for both questions regarding all procedures were calculated. We additionally performed subgroup analysis using cross tabulation and Chi-square test for evaluation of differences in the performance of most frequently performed procedures in urban or rural areas as well as by male or female physicians. Subgroups themselves were compared regarding sociodemographic details using Chi-square test, Mann-Whitney- or Student’s t-test in consideration of scale level and normal distribution of data. For all tests we referred to statistically significance with *p* < 0.05. In order to correct for multiple comparisons Bonferroni correction was used for the evaluation of differences regarding the most frequently performed procedures. Thus, each hypothesis was tested at α = 0.002.

### Ethics approval

The institutional review board (IRB) of the Heidelberg University Hospital informed on inquiry for the previous study with the same questionnaire, family physicians as participants and a similar course of action [18] that an anonymous survey would not need a vote by the IRB (correspondence from August 2, 2012). Therefore, an ethical approval regarding this following study was not obtained.

## Results

The overall response rate was 30% (302/1002), whereof 28% (276/1002) of incoming questionnaires could be included into analysis. In total, 26 participants with specialties other than internal medicine or with more than one specialty were excluded from analysis.

Participants were on average 52 years old with 13 years practice experience in primary care; 60% were male. In total, 46% participants stated that their practice was in a rural area although the population of practice location was stated to be more than 20,000 inhabitants by seven participants. For further details see Table 1.

**Table 1:** Participants‘ sociodemographic characteristics (*n* = 276)

		**N (%)**
Sex	Female	109 (39.5)
	Male	166 (60.1)
Practice location (with localities‘ population)	**Rural area**	**127 (46.0)**
	< 5000 inhabitants	45 (35.4)
	5000–10,000 inhabitants	40 (31.5)
	> 10,000–20,000 inhabitants	34 (26.8)
	> 20,000–50,000 inhabitants	5 (3.9)
	> 50,000–100,000 inhabitants	2 (1.6)
	> 100,000 inhabitants	0
	**Urban area**	**146 (52.9)**
	< 5000 inhabitants	1 (0.7)
	5000–10,000 inhabitants	4 (2.7)
	> 10,000–20,000 inhabitants	27 (18.5)
	> 20,000–50,000 inhabitants	51 (34.9)
	> 50,000–100,000 inhabitants	28 (19.2
	> 100,000 inhabitants	34 (23.3)
Population of practice location	< 5000 inhabitants	46 (16.7)
	5000–10,000 inhabitants	44 (15.9)
	> 10,000–20,000 inhabitants	63 (22.8)
	> 20,000–50,000 inhabitants	57 (20.7)
	> 50,000–100,000 inhabitants	30 (10.9)
	> 100,000 inhabitants	34 (12.3)
Practice model	Solo practice	122 (44.2)
	Practice with more than one physician	141 (51.1)
	Other	12 (4.3)
Average number of patients per quarter	< 500 patients	9 (3.3)
	> 500–1000 patients	52 (18.8)
	> 1000–1500 patients	100 (36.2)
	> 1500–2000 patients	40 (14.5)
	> 2000–2500 patients	34 (12.3)
	> 2500 patients	40 (14.5)
		**Mean (SD**^**a**^**) (Range)**
Age		52.5 (9.2) (32–81)
Years practiced in primary care		13.4 (9.3) (1–41)

^a^Standard deviation

**Fig. 1:**
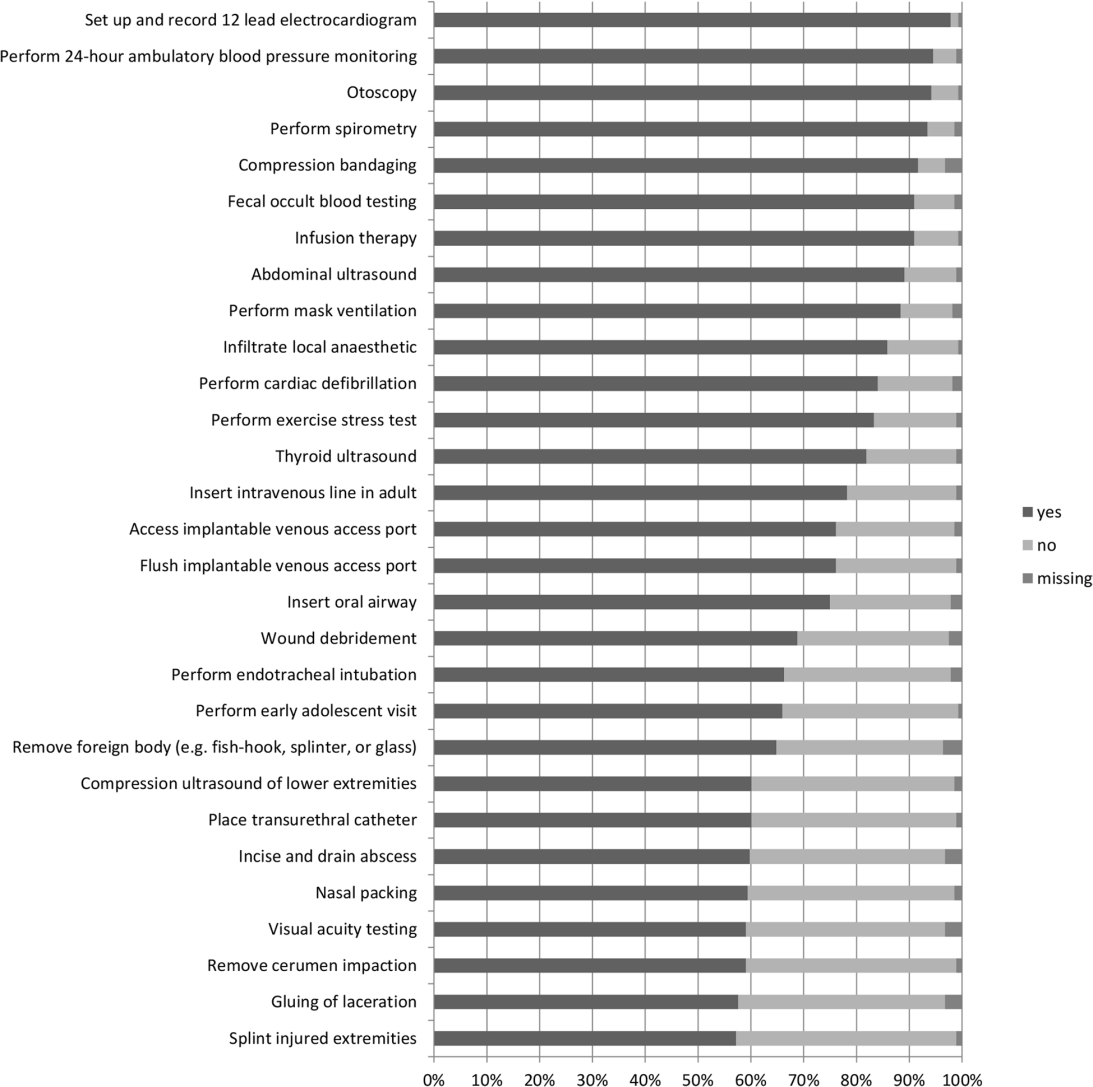
Procedures performed by at least 50% of participating general internists working in primary care

Overall, 29 procedures were performed by more than 50% of the participating general internists. These procedures belong to the areas technical diagnostics (seven out of nine procedures), punctures (five out of 14), integument (five out of 16), resuscitation (four out of five), ears (two out of five) as well as eyes (one out of eight), nose (one out of three), gastrointestinal tract (one out of seven), urogenital system (one out of seven), obstetrics and pediatrics (one out of four) and musculoskeletal system (one out of nine). Further details are displayed in Fig. 1.

**Fig. 2:**
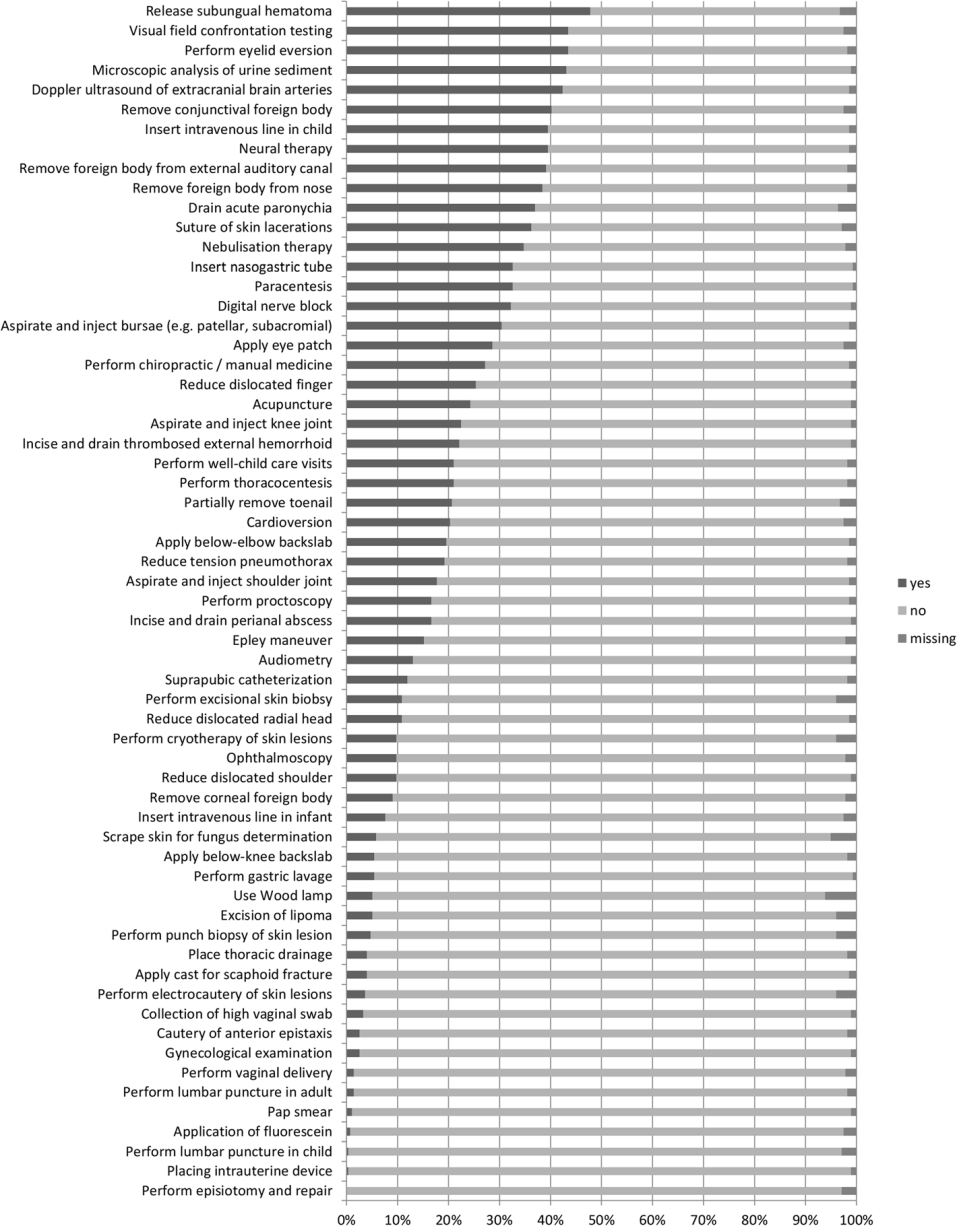
Procedures performed by less than 50% of participating general internists working in primary care

Least performed procedures were episiotomy and its suturing (0.0%), the placement of an intrauterine device (IUD, 0.4%) and lumbar punctures in children (0.4%). For further information see Fig. 2.

**Fig. 3:**
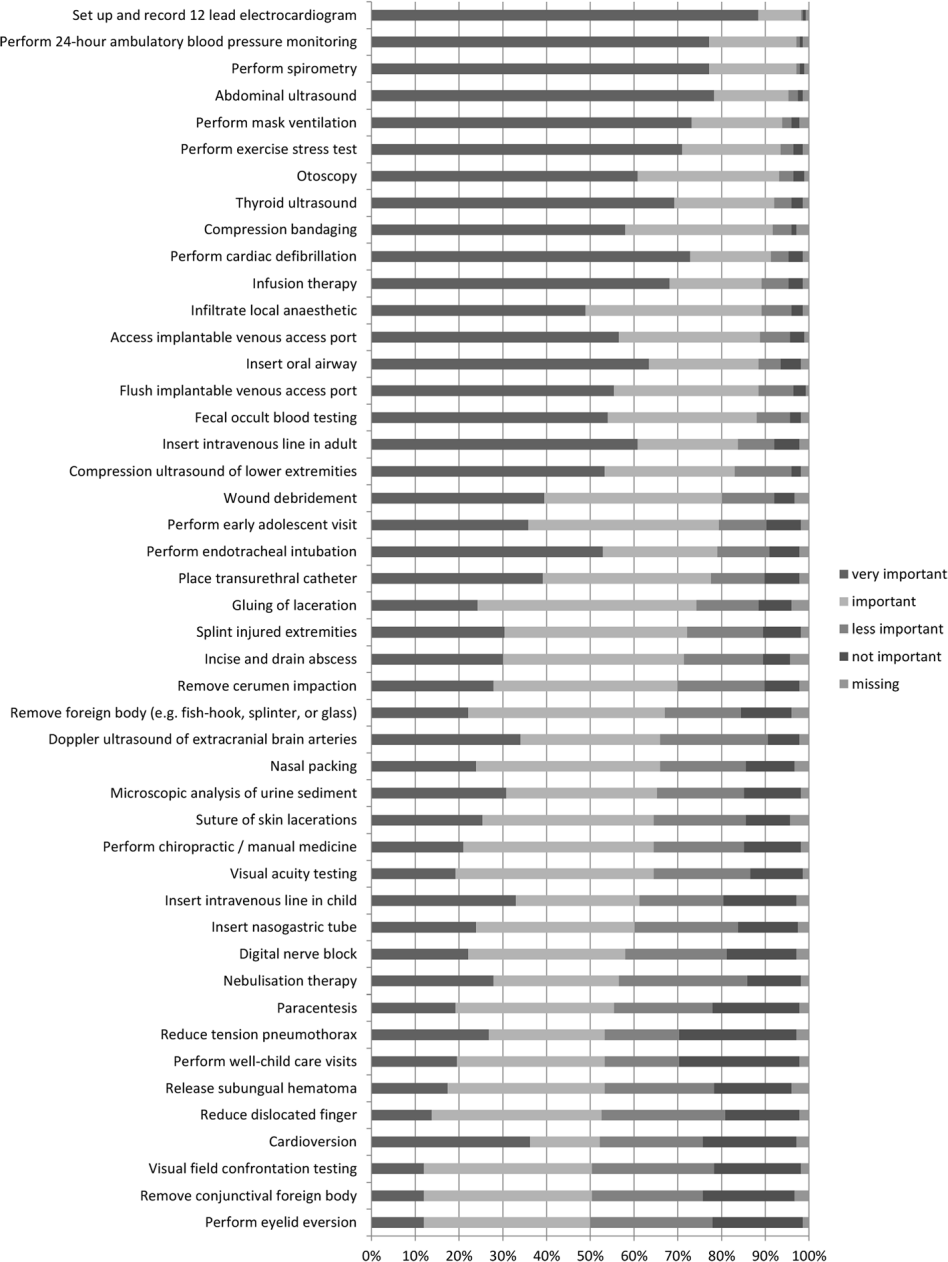
Procedures assessed as important to learn during residency by at least 50% of participants

Moreover, 46 procedures were assessed by more than 50% of the participants as “very important” or “important” to learn during residency. Figure 3 delivers more details.

**Fig. 4:**
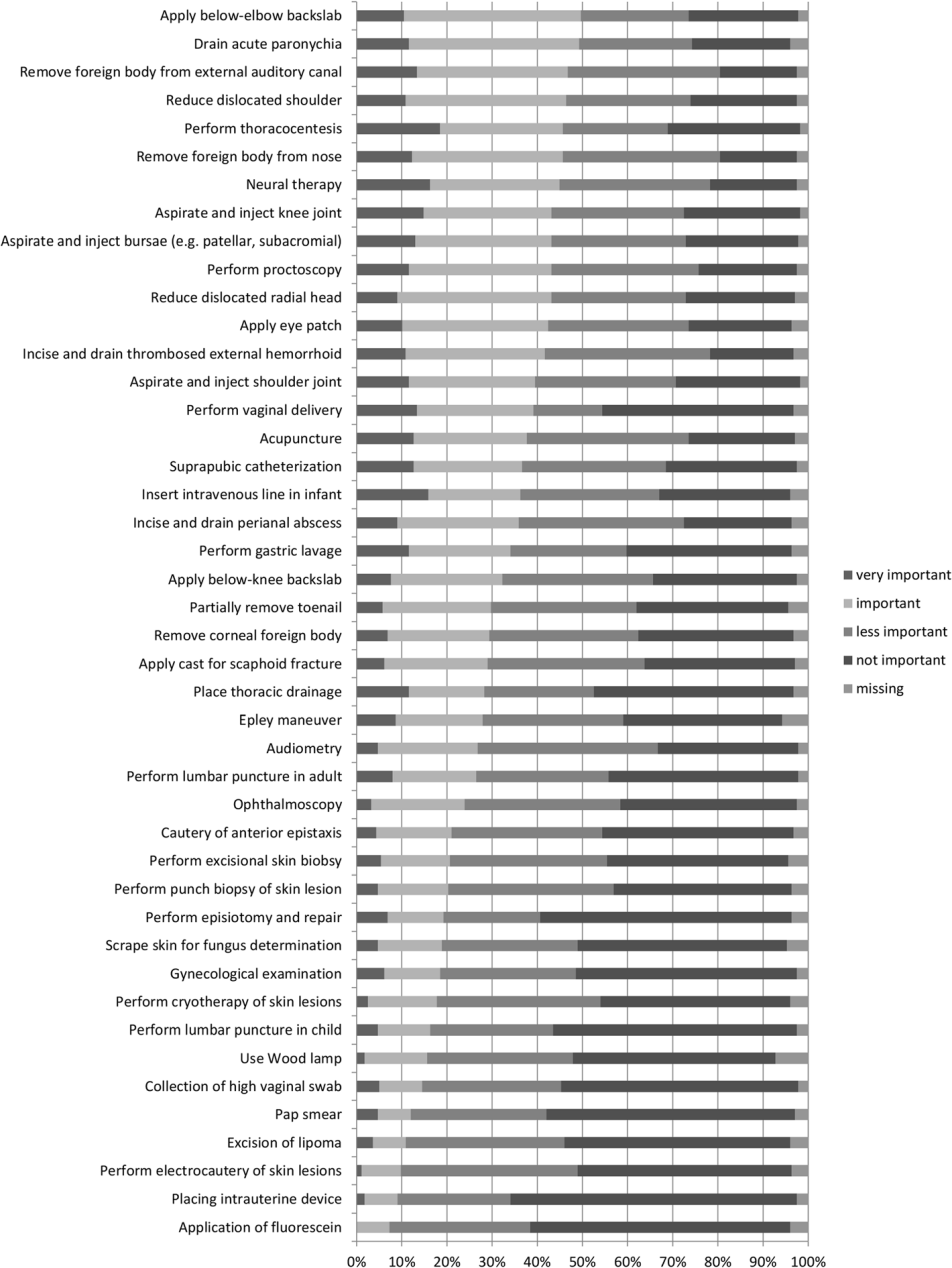
Procedures assessed as important to learn during residency by less than 50% of participants

Stated as least important to learn during residency were the application of fluorescein (7%), the placement of an IUD (9%) and the performance of electrocautery of skin lesions (10%). For more information see Fig. 4.

Subgroup analysis revealed that performance of the 29 most frequently performed procedures in this questionnaire differed regarding physician’s sex and practice location. Five procedures were performed by more male than female physicians. No procedures were performed more frequently by female physicians. More details are displayed in Table 2.

**Table 2:** Subgroup analysis of general internist’s sex and practice location

Procedure	Urban practice (***N*** = 146) % (N)	Rural practice (***N*** = 127)% (N)	***p*** value	Female physician (***N*** = 109) % (N)	Male physician (***N*** = 166% (N)	***p*** value
Access implantable venous access port	not statistically significant			32.4 (68)	**67.6 (142)**	< 0.001
Flush implantable venous access port	not statistically significant			32.9 (69)	**67.1 (141)**	< 0.001
Compression ultrasound of lower extremities	not statistically significant			31.9 (53)	**68.1 (113)**	0.001
Incise and drain abscess	not statistically significant			31.5 (52)	**68.5 (113)**	0.001
Nasal packing	45.4 (74)	**54.6 (89)**	0.001	not statistically significant		
Remove cerumen impaction	42.9 (69)	**57.1 (92)**	< 0.001	31.9 (52)	**68.1 (111)**	0.001

Procedures are listed according to the frequency of performance by participants, statistically significant numbers after Bonferroni correction are printed bold

The subgroups of male and female physicians did not differ in age (*p* = 0.191) or practice experience (*p* = 0.158), but did differ in practice model (*p* = 0.032) with male physicians working more frequently in practices with more than one physician. Male physicians also saw more patients in 3 months (*p* < 0.001) with, accordingly, a higher patient number stated by male physicians.

Two procedures were performed more frequently by physicians working in a rural practice than physicians practicing in an urban location (Table 2). Subgroups of rural and urban practicing physicians differed in their age (*p* = 0.015); physicians practicing in rural areas had a mean age of 64 years and those in urban areas had a mean age of 61 years. Practice experience did not differ statistically significantly (*p* = 0.088).

In the free text section, it was commented that some procedures are not performed in primary care due to missing financial incentives. Particularly surgical procedures, foremost included in the area integument in this questionnaire, would require investment in special devices that would not pay off.

## Discussion

We performed a cross sectional study surveying the procedural performance of general internists working in primary care in Germany. In total, 29 out of 90 provided procedures were performed by more than 50% of participating general internists. Performance differences could be attributed to physician’s sex and practice location.

With 276 general internists participating in this study, respondents represent about 2% of all practicing general internists working in primary care in Germany in 2015 [11]. Sociodemographic characteristics match official numbers with 61.6% male and 38.4% female general internists working in primary care in Germany with an average age of 53 years [11].

Procedures performed by most general internists were technical diagnostics and emergency procedures. A more technical style of care by general internists has already been described [19, 20].

Reasons for encounter in family medicine were shown to be internationally similar with 35 groups of reasons covering the thirty most frequently reasons for encounter in Dutch, Japanese and Polish family medicine and US ambulatory medical care, covering 70 to 75% of all encounters per 1000 patients per year [21]. The wide scopes of family medicine and US ambulatory care in general are underlined by the distribution of reasons for encounter showing respiratory, musculoskeletal, digestive, neurological, psychological, circulatory and urinary reasons as well as reasons regarding ears, eyes and skin [21, 22]. Correspondingly, a wide scope in procedures might be necessary in order to provide best possible care for patients’ complaints. A study with 871 diabetic patient visits showed that 2.6 procedures on average had been performed per visit in family medicine comprising diagnostic and therapeutic procedures belonging to a wide scope of areas including metabolic, circulatory, musculoskeletal and respiratory procedures [23].

The revisited Ecology of Care described that out of 1000 women, men and children in the US 113 would visit a primary care physician each month in contrast to eight being hospitalized [24]. Although primary care visits have declined significantly between 2002 and 2012 [25], it remains a big resource for delivering high quality care. One component of quality care was described by providing care of high technical quality: Procedures, tests or services are performed in a technically excellent manner only when “desired health outcomes exceed the health risks by a sufficiently wide margin” [26]. Due to this definition, it is not enough to train the most possible variety of procedures, but also know about risks, effects and the appropriate reasons to perform procedures that have been proven effective. By now, there are lists of core procedures in family medicine reached by group consensus in the US and by a two round Delphi process in Australia and Canada [15–17]. Matching with patients‘and health care systems‘needs is missing. Whether these core procedures have to be applied for general internists working in primary care has to be discussed in respect of individual health care systems. In Germany, patient spectrum and reasons for encounter are exchangeable for general internists working in primary care and family physicians. Patients know them both as “family doctors” and contact them with all their needs. That is why both specializations need to fulfill the same scope of practice and procedures in a qualitatively high manner.

In a multivariable model, factors were shown that predicted a broader scope of family physicians: male physician sex, being in group practice, greater access to hospital beds and less access to specialists [27]. This is reflected by our data for general internists as well. Male physicians perform 17% of the most frequently performed procedures significantly more frequently than their female colleagues. They work significantly more frequently in a practice with more than one physician. Group practices with a higher patient flow might allow maintaining adequate equipment for surgical procedures and technical diagnostics that otherwise would not be affordable with less patients requiring those procedures as indicated in the free text section of this study. In terms of character, the spectrum stated by male general internists seems to be more invasive. Despite similar clinical hours, a comparison between male and female ophthalmologists showed more surgical time by male physicians [28], whereas female physicians seeking board certification for family medicine tent to engage in more dialog with patients than their male counterparts [29]. Gender differences in practice style have to be taken into account in residency training programs in order to train what is expected from both sexes.

Seven percent of the most frequently performed procedures are more likely to be performed in a rural rather than an urban practice site, where access to specialists might be limited. Less broadly trained general internists may not work in a rural setting with missing referral possibilities, which they are less prepared for.

A greater emphasis on primary care and well trained generalists can reduce health costs, improve health through access to more appropriate services, and reduce the inequities in the population’s health and mortality leading to better health outcomes [30, 31].

Comprehensively structured primary care is seriously needed. Strategies proposed to sharpen the status of general internists working in primary care must be realized [20, 32, 33] in order to prevent further deficits in performance quality and physician quantity. Additional structured post residency training as one mean of broadening the scope has already been identified [27], and can be an opportunity to fill gaps after board certification.

### Strengths and limitations

This cross-sectional study was performed by measuring the self-assessments of general internists working in German primary care. With a statistical sample of 2%, the generalizability cannot be granted although sociodemographic characteristics match the total population of general internists in Germany.

Moreover, we cannot assure that results reflect whether procedures are really performed by participants. Low prevalence procedures might be crossed as “Not performed in own practice”, although participants could perform the procedure when necessary. Otherwise, participants might have stated all procedures they were able to perform as “Performed in own practice”, although they had never the opportunity to perform those procedures in reality.

Furthermore, this cross-sectional study only shows associations between the performance of procedures with practice region and physician’s sex; causal links cannot be drawn. Revealed associations have to be investigated in further studies.

In this context, it is important to notice that women tend to self-assess their competencies more cautiously than men [34–36]. Thus, gender differences in self-assessed procedural performance might also be related to this reserved response behavior. Objective measurement is needed to clarify this.

Since the majority of participants worked in a group practice, the procedural scope displayed here might be skewed by asking only one physician or the physicians separately. The direct influences of group practices (between general internists, general internists and family physicians or general internists and other specialties like pediatrics) on procedural scope should be investigated further.

Additionally, the frequency of procedural performance by each participant was not surveyed. However, this attribute might be conclusive in revealing why procedures are performed by more or less general internists, and their importance for residency training. This aspect should be investigated by further research in order to name core procedures in general internal medicine.

Finally, previous studies in other countries showed diverse focuses of procedural performance in primary care especially regarding women’s reproductive health performing pap smear and IUD placement [16, 17]. In Germany, contraceptive measures are mostly introduced by gynecologists [37]. Core procedures differ between countries and must be adapted according to the existing health care system.

## Conclusions

General internists working in German primary care perform a narrow scope of procedures offered by primary care physicians. In order to provide best ambulatory care for patients, residency training programs must ensure training in procedures that are necessary for providing high quality care. Therefore, a consensus aligned with patients’ and health-systems’ needs on procedures required for working as a general internist in primary care is necessary.

## Supplementary information

**Additional file 1.** Questionnaire “Procedures in Primary Care”

## Abbreviations

WHO: World Health Organization
ASHIP: Association of Statutory Health Insurance Physicians
IUD: Intrauterine Device
