# Supplementary material for: Which procedures are performed by general internists practicing primary care in Germany? - a cross-sectional study
Source: BMC Fam Pract. 2020 Apr 29;21:73. doi: 10.1186/s12875-020-01136-7 (PMC7191754; doi:10.1186/s12875-020-01136-7)
Supplement: Supplementary file 1 — Additional file 1. Questionnaire “Procedures in Primary Care” [file 12875_2020_1136_MOESM1_ESM.pdf]

Procedures in Primary Care

If not indicated otherwise, please choose one single answer. We define procedures in the following as self-contained, diagnostic or therapeutic activities performed on patients, which require knowledge and manual skills.

Please tick **two boxes per line** in the following part of the questionnaire.

Procedures	How important do you think it is to learn this procedure during residency?					Do you perform this procedure in your own practice?	
	very important	important	less important	not important		yes	no
**Integument**							
1. Incise and drain abscess	☐	☐	☐	☐		☐	☐
2. Wound debridement	☐	☐	☐	☐		☐	☐
3. Suture of skin lacerations	☐	☐	☐	☐		☐	☐
4. Gluing of laceration	☐	☐	☐	☐		☐	☐
5. Perform punch biopsy of skin lesion	☐	☐	☐	☐		☐	☐
6. Perform excisional skin biopsy	☐	☐	☐	☐		☐	☐
7. Excision of lipoma	☐	☐	☐	☐		☐	☐
8. Perform cryotherapy of skin lesions	☐	☐	☐	☐		☐	☐
9. Perform electrocautery of skin lesions	☐	☐	☐	☐		☐	☐
10. Scrape skin for fungus determination	☐	☐	☐	☐		☐	☐
11. Use Wood lamp	☐	☐	☐	☐		☐	☐
12. Release subungual hematoma	☐	☐	☐	☐		☐	☐
13. Drain acute paronychia	☐	☐	☐	☐		☐	☐
14. Partially remove toenail	☐	☐	☐	☐		☐	☐
15. Remove foreign body (e.g. fish-hook, splinter, or glass)	☐	☐	☐	☐		☐	☐
16. Compression bandaging	☐	☐	☐	☐		☐	☐

Procedures	How important do you think it is to learn this procedure during residency?				Do you perform this procedure in your own practice?	
	very important	important	less important	not important	yes	no
**Eyes**						
17. Visual acuity testing	☐	☐	☐	☐	☐	☐
18. Visual field confrontation testing	☐	☐	☐	☐	☐	☐
19. Perform eyelid eversion	☐	☐	☐	☐	☐	☐
20. Application of fluorescein	☐	☐	☐	☐	☐	☐
21. Ophthalmoscopy	☐	☐	☐	☐	☐	☐
22. Remove conjunctival foreign body	☐	☐	☐	☐	☐	☐
23. Remove corneal foreign body	☐	☐	☐	☐	☐	☐
24. Apply eye patch	☐	☐	☐	☐	☐	☐
**Ears**						
25. Otoscopy	☐	☐	☐	☐	☐	☐
26. Remove cerumen impaction	☐	☐	☐	☐	☐	☐
27. Audiometry						
28. Remove foreign body from external auditory canal	☐	☐	☐	☐	☐	☐
29. Epley maneuver	☐	☐	☐	☐	☐	☐
**Nose**						
30. Remove foreign body from nose	☐	☐	☐	☐	☐	☐
31. Cautery of anterior epistaxis	☐	☐	☐	☐	☐	☐
32. Nasal packing	☐	☐	☐	☐	☐	☐
**Chest**						
33. Perform thoracocentesis	☐	☐	☐	☐	☐	☐
34. Reduce tension pneumothorax	☐	☐	☐	☐	☐	☐
35. Place thoracic drainage	☐	☐	☐	☐	☐	☐

Procedures	How important do you think it is to learn this procedure during residency?					Do you perform this procedure in your own practice?	
	very important	important	less important	not important		yes	no
**Gastrointestinal Tract**							
36. Insert nasogastric tube	☐	☐	☐	☐		☐	☐
37. Perform gastric lavage	☐	☐	☐	☐		☐	☐
38. Fecal occult blood testing	☐	☐	☐	☐		☐	☐
39. Perform proctoscopy	☐	☐	☐	☐		☐	☐
40. Incise and drain thrombosed external hemorrhoid	☐	☐	☐	☐		☐	☐
41. Incise and drain perianal abscess	☐	☐	☐	☐		☐	☐
42. Paracentesis	☐	☐	☐	☐		☐	☐
**Urogenital System**							
43. Microscopic analysis of urine sediment	☐	☐	☐	☐		☐	☐
44. Place transurethral catheter	☐	☐	☐	☐		☐	☐
45. Suprapubic catheterization	☐	☐	☐	☐		☐	☐
46. Gynecological examination	☐	☐	☐	☐		☐	☐
47. Collection of high vaginal swab	☐	☐	☐	☐		☐	☐
48. Pap smear	☐	☐	☐	☐		☐	☐
49. Placing intrauterine device	☐	☐	☐	☐		☐	☐
**Obstetrics and Pediatrics**							
50. Perform vaginal delivery	☐	☐	☐	☐		☐	☐
51. Perform episiotomy and repair	☐	☐	☐	☐		☐	☐
52. Perform well-child care visits	☐	☐	☐	☐		☐	☐
53. Perform early adolescent visit	☐	☐	☐	☐		☐	☐

Procedures	How important do you think it is to learn this procedure during residency?					Do you perform this procedure in your own practice?	
	very important	important	less important	not important		yes	no
**Musculoskeletal System**							
54. Splint injured extremities	☐	☐	☐	☐		☐	☐
55. Reduce dislocated finger	☐	☐	☐	☐		☐	☐
56. Reduce dislocated radial head	☐	☐	☐	☐		☐	☐
57. Reduce dislocated shoulder	☐	☐	☐	☐		☐	☐
58. Apply below-elbow backslab	☐	☐	☐	☐		☐	☐
59. Apply cast for scaphoid fracture	☐	☐	☐	☐		☐	☐
60. Apply below-knee backslab	☐	☐	☐	☐		☐	☐
61. Aspirate and inject bursae (e.g. patellar, subacromial)	☐	☐	☐	☐		☐	☐
62. Perform chiropractic / manual medicine	☐	☐	☐	☐		☐	☐
**Resuscitation**							
63. Insert oral airway	☐	☐	☐	☐		☐	☐
64. Perform mask ventilation	☐	☐	☐	☐		☐	☐
65. Perform endotracheal intubation	☐	☐	☐	☐		☐	☐
66. Perform cardiac defibrillation	☐	☐	☐	☐		☐	☐
67. Cardioversion	☐	☐	☐	☐		☐	☐
**Punctures**							
68. Infiltrate local anaesthetic	☐	☐	☐	☐		☐	☐
69. Digital nerve block	☐	☐	☐	☐		☐	☐
70. Aspirate and inject knee joint	☐	☐	☐	☐		☐	☐
71. Aspirate and inject shoulder joint	☐	☐	☐	☐		☐	☐

Procedures	How important do you think it is to learn this procedure during residency?					Do you perform this procedure in your own practice?	
	very important	important	less important	not important		yes	no
72. Insert intravenous line in adult	☐	☐	☐	☐		☐	☐
73. Insert intravenous line in child	☐	☐	☐	☐		☐	☐
74. Insert intravenous line in infant	☐	☐	☐	☐		☐	☐
75. Perform lumbar puncture in adult	☐	☐	☐	☐		☐	☐
76. Perform lumbar puncture in child	☐	☐	☐	☐		☐	☐
77. Infusion therapy	☐	☐	☐	☐		☐	☐
78. Access implantable venous access port	☐	☐	☐	☐		☐	☐
79. Flush implantable venous access port	☐	☐	☐	☐		☐	☐
80. Acupuncture	☐	☐	☐	☐		☐	☐
81. Neural therapy	☐	☐	☐	☐		☐	☐
**Technical Diagnostics**							
82. Set up and record 12 lead electrocardiogram	☐	☐	☐	☐		☐	☐
83. Perform exercise stress test	☐	☐	☐	☐		☐	☐
84. Perform 24-hour ambulatory blood pressure monitoring	☐	☐	☐	☐		☐	☐
85. Perform spirometry	☐	☐	☐	☐		☐	☐
86. Abdominal ultrasound	☐	☐	☐	☐		☐	☐
87. Thyroid ultrasound	☐	☐	☐	☐		☐	☐
88. Compression ultrasound of lower extremeties	☐	☐	☐	☐		☐	☐
89. Doppler ultrasound of extracranial brain arteries	☐	☐	☐	☐		☐	☐
90. Nebulisation therapy	☐	☐	☐	☐		☐	☐

Sociodemographic Data

91. What year were you born? Please enter the date.

19|__|__|

92. Your sex? ☐ female ☐ male

93. For how many years have you been practicing in primary care (after board certification)? |__|__| Jahre

94. If you were board certified before 1990: Where did you complete your residency?

☐ Newly-formed German states ☐ Former West German states ☐ Residency after 1990

95. Where is your (main) practice location?

☐ Rural area ☐ Urban area

96. How many inhabitants does your practice location have?

☐ < 5,000 inhabitants ☐ 5,000 - 10,000 inhabitants

☐ > 10,000 - 20,000 inhabitants ☐ > 20,000 - 50,000 inhabitants

☐ > 50,000 - 100,000 inhabitants ☐ > 100,000 inhabitants

97. Which specialty do you belong to?

☐ General Practitioner ☐ Specialist in Family Medicine

☐ Specialist in Internal Medicine ☐ Other: ______________________

98. What is your practice model?

☐ Solo practice ☐ Practice with more than one physician

☐ Other: ____

99. What is the average number of patients per quarter in your practice? Please count patients with statutory **and** private health insurance.

☐ < 500 patients ☐ > 500 - 1,000 patients

☐ > 1,000 - 1,500 patients ☐ > 1,500 - 2,000 patients

☐ > 2,000 - 2,500 patients ☐ > 2,500 patients

100. If you think procedures are missing in this questionnaire, please add them here:

Thank you very much for your participation!
